# Exploring Intervention Frameworks to Improve Utilization of Elimination of Mother-to-Child Transmission Services in Africa: A Scoping Review

**DOI:** 10.3390/nursrep14030190

**Published:** 2024-09-23

**Authors:** Ndivhuwo Mukomafhedzi, Takalani Tshitangano, Shonisani Tshivhase

**Affiliations:** 1 Department of Public Health, Faculty of Health Sciences, University of Venda, Thohoyandou 0950, South Africa; 11560403@mvula.univen.ac.za; 2 Department of Public Health, Faculty of Health Care Sciences, University of Limpopo, Polokwane 0727, South Africa

**Keywords:** EMTCT services, nursing mothers, pregnant women, utilization

## Abstract

Background: Over the past two decades, intervention strategies to improve the use of the elimination of mother-to-child transmission (EMTCT) services have been implemented for several reasons. The reasons include elimination of HIV infections during pregnancy, delivery, breastfeeding, prevention of HIV, prevention of unintended pregnancies, and safer conception. Poor utilization of EMTCT services has been proven to put the child at risk of acquiring HIV, which could have been avoided. Objective: This study aims to explore and describe interventions to promote the elimination of mother-to-child transmission services among pregnant and nursing mothers in Africa. Method: A scoping literature review technique was undertaken on research papers published in English that focused on EMTCT, barriers, interventions, and methods to address challenges to EMTCT utilization. These were screened independently and coded. Results: The analysis comprised 14 out of approximately 9029 literature sources. Intervention strategies to improve EMTCT service utilization, according to the findings, include accessibility and affordability, healthcare worker training, integrating the elimination of mother-to-child transmission into maternal and child health services, community-based interventions, family-centred approaches, and the use of technology. Conclusions: Interventions that increase women’s use of EMTCT services will contribute to the aim of HIV-free generation by reducing new HIV infections in children and saving lives.

## 1. Introduction

For more than two decades, the world has been working to prevent HIV transmission from mother to child (EMTCT) [1,2]. The World Health Organization (WHO) aimed towards stopping the spread of HIV from mothers to their babies by 2015, setting this goal in 1994 [3]. Although we did not reach the goal, we have made great improvements in lowering the number of new HIV infections in children.

In 2018, about 260,000 babies were born with HIV around the world, a decrease from 330,000 who were born in 2010 [4]. However, EMTCT rates vary greatly between countries and locations. Sub-Saharan Africa has the most cases of HIV and the highest rates of preventing mother-to-child transmission. In 2018, about 170,000 babies in Sub-Saharan Africa were born with HIV, making up 65% of all new HIV cases [3,4].

The first intervention frameworks for improving EMTCT service utilization were created in the early 1990s [1,5,6]. These frameworks aimed to raise awareness of HIV preventive services while also lowering structural access hurdles. In the late 1990s, a variety of innovative intervention approaches emerged that tackled stigma and discrimination [4].

The “ABC” strategy for HIV prevention focuses on avoiding sex, preserving a partner’s loyalty, and making use of condoms [7]. The ABC technique was initially created to prevent HIV transmission in adults, but it was later adapted to prevent vertical transmission. The ABC strategy was incorporated into a number of national HIV prevention efforts in Africa, and it helped to reduce the incidence of new HIV infections among adults [7,8]. However, the ABC method was ineffective in preventing mother-to-child transmission, necessitating the development of other intervention strategies. In the mid-2000s, a variety of new intervention frameworks were created to improve EMTCT service usage in Africa [9,10]. These frameworks were built on the concept of “Option B+”, a strategy that advocates for all pregnant women, regardless of HIV status, to receive lifelong ART. Option B+ has been demonstrated to be highly effective in preventing vertical transmission of HIV transmission, and it is now the preferred method of preventing EMTCT in the majority of nations. According to the study conducted in South Africa, the Option B+ approach reduced the rates of vertical transmission of HIV by 95% [1,3,4,11].

Antiretroviral therapy (ART), first introduced in HIV-positive pregnant women in 2004, marked a watershed moment. This discovery dramatically reduced transmission rates and improved maternal health outcomes [1]. Additionally, efforts have been made nationwide to integrate EMTCT services into existing mother-and-child health programmes at basic healthcare facilities.

Despite the availability of effective EMTCT interventions, there are considerable hurdles to their use in Africa. These barriers can be encountered at the individual, community, and healthcare system levels. Individual challenges included stigma, fear of discrimination, lack of awareness of EMTCT, and concerns about adverse treatment effects. Barriers at the community level include lack of access to EMTCT services, cultural beliefs that discourage women from seeking HIV testing and treatment, and societal conventions that discourage breastfeeding. Inadequate training of health personnel, a lack of resources, and long wait periods for services are examples of health system-level impediments.

Isindu et al. and Vieira et al. [12,13] stated that inadequate knowledge about EMTCT services among healthcare practitioners and patients is a key barrier to offering comprehensive services. This knowledge gap results in missed opportunities for early HIV diagnosis and treatment, thereby increasing mother-to-child transmission rates [14]. Adolescents and young women are particularly affected, and their lack of understanding makes it difficult to access EMTCT services. Moreover, according to Kamanzi and Ritcher; Teshale et al. [15,16], many women in developing countries are unaware of HIV and AIDS prevention programmes because of their inadequate healthcare infrastructure, limited knowledge, and cultural attitudes. This results in the virus being transmitted unwittingly to children. Ramoshaba and Sithole [17] argued that most women are aware of EMTCT services, but they lack knowledge about pregnancy, birth, and HIV transmission during the nursing period. Thus, there is a need to conduct ongoing training on current guidelines for healthcare workers and patients.

Long waiting times and distances to healthcare facilities hinder women’s access to EMTCT services, with stigma and a lack of transportation causing frustration and dissatisfaction [18,19]. Sidze et al. [20] commented that geographic location significantly impacts access to EMTCT services for pregnant and nursing mothers in rural or remote areas. Improving accessibility is crucial for ending new HIV infections in children [19,21]. Strategies include appointment scheduling, triage systems, community-based testing counselling, mobile clinics, and task shifting. EMTCT services at government institutions are free and accessible to pregnant women and lactating mothers, regardless of age, nationality, beliefs, marital status, or socioeconomic status [22]. These services should be provided respectfully, non-judgmentally, and equitably, following primary healthcare principles.

The current review attempts to determine the appropriate type of intervention that can be put in place to promote the uptake of EMTCT services in Africa to reduce the vertical transmission of HIV. This can be achieved by the provision of education on EMTCT services, community involvement, and a family-centered approach.

## 2. Materials and Methods

A rigorous database search was conducted. The PRISMA-ScR (Preferred Reporting Items for Systematic Reviews and Meta-Analyses Extension for Scoping Reviews) standards and recommendations were used to examine the current methodological frameworks and barriers to EMTCT service utilization. The scoping review, created following the approach proposed by Arksey and O’Malley, seeks to identify areas of interest and potential research needs [23].

### 2.1. Identifying the Research Questions

There has been a significant gap in EMTCT service utilization among pregnant and nursing mothers, which has revealed a huge gap in access and retention of EMTCT services. Even though strides have been made to improve EMTCT service utilization, gaps hinder these women from accessing the service. Notably, inadequate knowledge, stigma, long waiting times, and distance from the facility cause frustrations and dissatisfaction [18,19,20]. This has prompted the researcher to look further into the literature and review possible interventions that might be employed to enhance their utilization of services. Even though several strategies have been used to improve EMTCT service utilization, utilization of these services remains poor in Africa. A scoping review technique was used to ensure a full and extensive analysis of the existing evidence, which is vital for designing successful strategies to promote the adoption of EMTCT services in Africa.

### 2.2. Identifying Relevant Studies

A comprehensive literature search was performed using numerous databases, including PubMed, EBSCOhost, Google Scholar, Sabinet, ProQuest, and reports on EMTCT/MTCT. A scoping literature review was conducted utilizing the population, intervention, comparison, and outcome (PICOs) framework. Keywords were used to find relevant publications [24]. Keywords were used to extract studies from each database. To acquire better literature, the field tag was from 2009 to 2023, written in English. This time frame is timeliness and relevant; this period allows for the inclusion of studies that reflect the influence of advances on EMTCT services.

### 2.3. Search Terms

Search terms were created based on this study objectives. Studies were chosen based on their similarities with the search term-referenced “EMTCT”, “EMTCT”, “women” “Africa”, and “EMTCT interventions”.

EMTCT search term was “EMTCT programme”

EMTCT search term was “EMTCT services”

Women-related search terms were “Pregnant” OR “ lactating”

Africa-related search terms were “Sub-Saharan Africa”, OR “Central Africa”, OR “Western Africa”, OR “Eastern Africa”, OR “North Africa”.

EMTCT intervention search terms were “support”, OR “education”, OR “accessibility”, OR “integration”

The use of quotation marks was consistent throughout the search database to retrieve studies with the exact terms in the titles, abstracts, and keywords presented in the paper.

### 2.4. Study Selection

**Figure 1:**
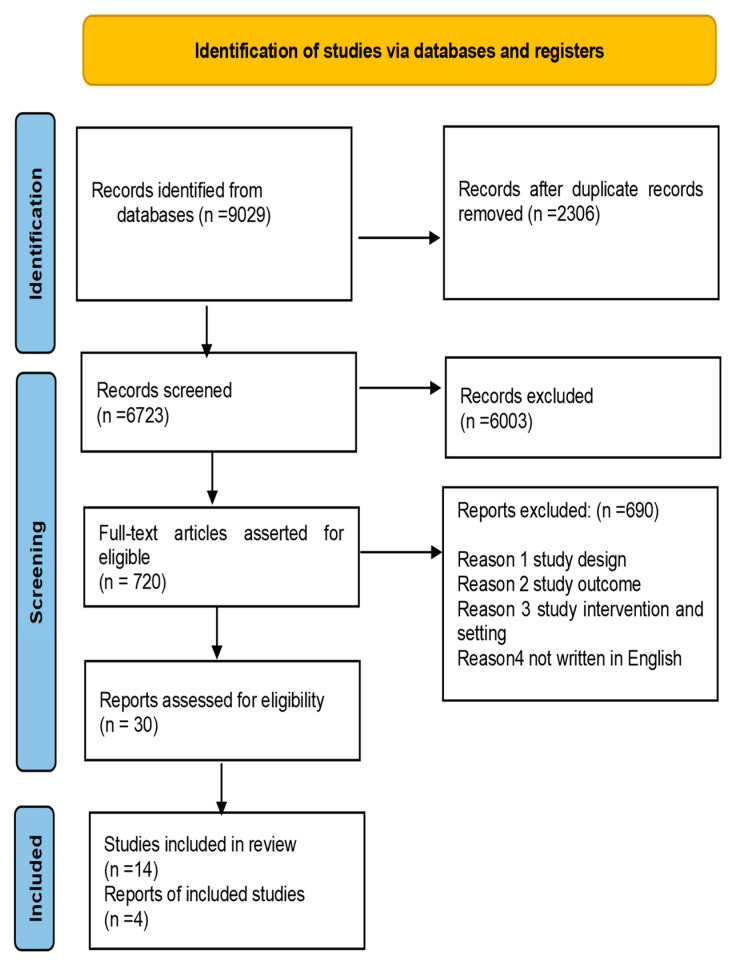
Flow diagram for inclusion criteria.

The researcher screened the articles by topic, abstract, and full text from the search engine. Duplicate studies and those not meeting the inclusion criteria were eliminated. A scoping literature review was carried out utilizing the population, intervention, comparison, and outcome (PICOs) framework, with keywords used to find relevant articles. The scoping review included only papers that followed the PICO approach, where the study assessed the intervention programmes on barriers to EMTCT service outcomes. Studies were selected if they described how an intervention affected the use of EMTCT services in Africa (Figure 1). The researcher excluded papers in which complete articles were unavailable. The researcher excluded studies that did not include the parts listed as preferred reporting items on the PRISMA-ScR checklist (Table 1) [25]. The literature sources in languages other than English were excluded. This study did not include unpublished research publications, conference abstracts, or presentations. The qualities of the included studies following the systematic appraisal of quality in observing research (SAQOR) (Table 2). 

### 2.5. Charting the Data

The researcher extracted data by comparing the information that was collected. As a result, for all selected studies that met the inclusion criteria, the following procedures were used to outline the characteristics of the included studies: the study’s country of origin, year of publication, study design, study objective, study population, sample size, study outcome, intervention, and control description (Table 3).

### 2.6. Collating, Summarizing, and Reporting Results

To extract data relevant to the review aims, a descriptive literature analysis was performed. Studies that lacked the primary component of the research objectives were removed. Quantitative data synthesis included gathering information from similar research and comparing it to the context in which it was conducted. Qualitative data were sorted and coded, and a coding scheme was developed expressly for this study. The produced codes and their units of meaning were topically evaluated to identify key themes. Tables and themes from various literatures were used to conduct the analysis. All pertinent facts were then summarized in tabular format, the characteristics of the selected studies (Table 2).

### 2.7. Critical Appraisal of the Review

The review of intervention frameworks to improve EMTCT service utilization is a valuable contribution to the field. The review gives a detailed summary of the evidence on the efficacy of various intervention tactics, as well as identification of a number of critical success elements. However, the review also has some limitations. The review gives a detailed summary of the evidence on the efficacy of various intervention tactics, as well as the identification of a number of critical success elements. First, the review is constrained by the quality of the available evidence. Many of the studies included in the evaluation were small, with methodological limitations. This makes it difficult to draw definitive conclusions about the efficacy of various intervention measures. The review is limited by its focus on a narrow range of intervention strategies. The review only included studies that focused on strengthening health systems, addressing social and cultural barriers, and involving communities. This means that the review does not provide an overview of the full range of interposition approaches that are available to enhance the utilization of EMTCT services.

Despite these limitations, the review provides valuable insights into the factors that are essential for improving EMTCT service utilization. The review highlights the importance of strengthening health systems, addressing social and cultural barriers, and involving communities in the design, implementation, and monitoring of EMTCT programs. These findings can help guide the development and implementation of more effective EMTCT programs.

## 3. Results

The review gives a detailed summary of the evidence on the efficacy of various intervention tactics, as well as the identification of a number of critical success elements. The search engines produced 9029 literature sources. After removing 2306 duplicates, there were 6723 items remaining, which were further removed 6003 times. After the abstract screening, 720 articles were maintained for full-text reading, whereas 680 articles were deleted for the following reasons: not written in English, duplicates, study design not related, study outcomes unrelated, study intervention, and setting. We chose 30 articles. Finally, 14 studies were deemed to be appropriate for this review because they met the inclusion criteria and responded to this study’s review question and purpose, as shown in Table 2. Figure 1 depicts fourteen source studies that demonstrated substantial results in intervention programs on barriers to EMTCT service outcomes. This section will classify topics that arose from the literature sources used in the scoping literature study. Six themes were developed from the selected articles included in this study regarding interventions to promote the utilization of EMTCT in Africa. The following themes were developed: accessibility and affordability of EMTCT services, healthcare worker training, integration of EMTCT into maternal and child health (MCH) services, community-based interventions, family-centered approaches, and the use of technology.

### 3.1. Theme 1: Accessibility and Affordability of EMTCT Services

EMTCT interventions are critical for reducing the incidence of new HIV infections in children [26]. However, access and affordability continue to be significant hurdles in the use and retention of these services. EMTCT services are neither easily accessible nor affordable in low-income countries, resulting in low uptake and retention rates [27,28]. This is exacerbated by the fact that many women do not have access to transportation and cannot afford to travel long distances to receive medical care. Additionally, cultural hurdles such as stigma and discrimination may hinder women from receiving EMTCT services [27].

Accessibility can be improved by expanding the number of health facilities offering EMTCT services, especially in remote regions. Additionally, community-based techniques such as mobile clinics and home-based care can boost access to EMTCT services [27]. Affordability can be addressed by providing free or subsidized EMTCT services for women with HIV. It is critical to make EMTCT services accessible and inexpensive to increase their utilization and retention. This can be accomplished by increasing funding for EMTCT programmes, improving infrastructure and staffing in health facilities, providing free or subsidized HIV testing and treatment for pregnant women, and implementing community-based interventions to raise awareness about the importance of EMTCT.

### 3.2. Theme 2: Healthcare Worker Training

EMTCT training for healthcare workers is critical for increasing the utilization and retention of this important healthcare service [1,27]. EMTCT aims to prevent HIV transmission from mother to child throughout pregnancy, birth, and nursing. EMTCT has been proven to greatly reduce the risk of vertical transmission of HIV, but its success is dependent on healthcare personnel’s knowledge and competence [29]. Healthcare worker training programmes should emphasize the need for early identification and treatment of HIV in pregnant women as well as effective counselling approaches for HIV-positive mothers [29]. They should also be instructed on how to properly administer and monitor antiretroviral medication (ART) [1]. Furthermore, training should address concerns about stigma and prejudice against HIV-positive individuals.

The effectiveness of this strategy is dependent on the availability and accessibility of high-quality healthcare services, which can only be delivered through adequate training of healthcare workers [29]. Healthcare personnel play a vital role in ensuring that pregnant women living with HIV receive proper care and treatment to prevent transmission to their babies [29]. They must be equipped with the information and abilities needed to offer comprehensive care during pregnancy, birth, and the postnatal period. This will lead to increased utilization and retention of EMTCT services, resulting in better health outcomes for both mothers and children. Furthermore, it will aid in ensuring that pregnant women living with HIV receive adequate care to protect both. Finally, continual training and assistance are required for the healthcare staff to maintain their skills and knowledge. This included regular supervision, mentoring, and ongoing education.

### 3.3. Theme 3: The Integration of EMTCT into MCH Services

Integration of EMTCT services into existing MCH programmes is a critical step in achieving the aim of eliminating mother-to-child HIV transmission [27]. During pregnancy, delivery, or nursing period, EMTCT services attempt to prevent vertical transmission of HIV. MCH programmes provide healthcare services to mothers and children, such as antenatal care, immunization, and nutritional advice. Integrating EMTCT services into MCH programmes can enable pregnant women living with HIV to access these critical services [27,30]. This also ensures that women receive thorough care during and after pregnancy.

Integrating EMTCT services into MCH programmes will assist in expanding access to these critical services for HIV-positive pregnant women [27,30]. Furthermore, integrating EMTCT services into existing MCH programmes can help reduce the stigma associated with HIV by normalizing the provision of these services within routine healthcare settings [30]. This method may help improve coordination among various healthcare providers involved in delivering care to pregnant HIV-positive women.

### 3.4. Theme 4: Community-Based Interventions

Community-based interventions have emerged as a promising approach to improving EMTCT services in Africa [31,32,33]. Marcos et al. and Besada et al. [31,34] have shown that these interventions improved several critical outcomes, including the increased uptake of antenatal care, HIV testing, and adherence to antiretroviral therapy. One of the most effective strategies identified was the use of community health workers (CHWs) to deliver EMTCT services. These individuals were trained to provide counselling, education, and support to pregnant women living with HIV. Their involvement significantly improved their knowledge about EMTCT, reduced the stigma associated with HIV/AIDS, and facilitated access to healthcare services [31,33].

Additionally, community mobilization activities, such as peer support groups and community dialogues were discovered to be useful in promoting consciousness and acceptance of EMTCT services [31,33,34]. These initiatives have created a supportive background for antenatal women living with HIV and encouraged them to seek timely care. However, challenges have been identified in the implementation of community-based interventions. These included limited resources for the educating and guiding of CHWs, inadequate infrastructure for service delivery at the community level, and cultural beliefs that hindered access to EMTCT services [32].

The most effective interventions included the following:

- Peer education and support: HIV-positive peer educators offer support and information to pregnant women and moms living with HIV. They also help reduce stigma and discrimination [34].
- Community mobilization: Community mobilization initiatives promote knowledge of EMTCT services and encourage women living with HIV to use them [33].
- Home-based care: Home-based care aids and monitors women living with HIV who are unable to attend health facilities [1,34]. Peer education and support: HIV-positive peer educators can provide support and information to women living with HIV. They can also contribute to reducing stigma and discrimination [34].

The review also found that community-based interventions are more effective when they are integrated with other services, such as HIV counselling and testing, and when tailored to the specific needs of the community [31,33,34]. Community-based interventions are crucial in improving the utilization of EMTCT services in Africa. However, relevant stakeholders, including community leaders, community health workers, women living with HIV, and their family members, should be involved in improving utilization and retention in the programme.

### 3.5. Theme 5: Family-Centred Approaches

The family-centered approach improved service uptake and retention in EMTCT programmes. According to Nyondo et al. [35], these interventions helped address the social, cultural, and economic barriers that hinder HIV-positive pregnant and breastfeeding women from attending EMTCT services. They aided in the development of trust and rapport between HIV-positive women and their families, encouraging them to adhere to EMTCT therapy and care.

Incorporating partners and other family members into HIV testing, treatment, and care decisions is a key aspect of family-centered methods [35]. Women are more likely to be supported and encouraged to use EMTCT services if their husbands and other families are involved.

According to Nyondo et al. [35], men are considered the head of the family and decision-makers in Africa because male partners offer financial support. Antenatal care has always been viewed as the primary responsibility of women. Male participation in EMTCT programmes boosts uptake and reduces HIV infection in children [36,37].

However, there is a barrier because male involvement in EMTCT services is limited, resulting in HV nondisclosure [35,37]. According to the literature, women are vulnerable to HIV infection and financially dependent on their husbands, making it difficult to negotiate condom use. According to Nyondo et al. [35], a lack of male partner involvement leads to women dropping out of the programme, putting their children at risk of contracting HIV. As a result, male partner and family participation in EMTCT services is critical because participation in these visits allows them to become more aware of EMTCT services and provide emotional and practical support to their partners.

Furthermore, a family-centered approach offers advice on newborn feeding alternatives, ensures access to antiretroviral medicine for both mothers and children, and addresses difficulties associated with HIV disclosure within the family [36]. According to Kalembo et al. [36], family-centered interventions have been proven to promote retention in care. Women are more likely to adhere to treatment regimens and attend follow-up sessions if their partners and other family members are included throughout the process [35,36]. It also addresses the social determinants of health, such as encouraging open communication within families. These techniques guarantee that pregnant women living with HIV receive comprehensive treatment, resulting in better health outcomes [35].

### 3.6. Theme 6: Use of Technology

Kassaye, Ojo, and Okal et al. [38,39,40] demonstrated the use of technology to boost the utilization of EMTCT services. For example, Ojo [39] discovered that a mobile phone-based intervention was effective in improving HIV testing among pregnant South African women. The initiative sent pregnant women reminders about HIV testing and counselling as well as instructional materials about EMTCT. According to the study, the initiative increased HIV testing among pregnant women by 25%.

Okal et al. [40] discovered that technology can be cost-effective in increasing the adoption of EMTCT services. Kassaye [38] demonstrated that the expense of adopting technology to promote EMTCT service use was compensated by the savings associated with avoiding mother-to-child HIV transmission.

## 4. Discussion

This scoping review explored interventions to enhance EMTCT service utilization among pregnant and nursing mothers. The review focused on five themes: accessibility and affordability of EMTCT services, healthcare worker training, integration of EMTCT into MCH services, community-based interventions, family-centered approaches, and the use of technology. Even though there was primary health care (PHC) re-engineering and integration of EMTCT services in maternal and child health programmes, women are still experiencing stigma and discrimination, which results in poor utilization of EMTCT services [27]. HIV remains a stigmatizing disease in many parts of Africa. This can deter women from seeking HIV testing and counselling and can also make it hard for them to seek EMTCT services [30]. Studies have revealed that healthcare workers are the main reason that hinders pregnant and nursing mothers’ use of these services due to negative staff attitudes and incompetent health personnel [18,19].

The use of EMTCT services has increased since the integration of EMTCT into the MCH programme; however, retention post-delivery remains a stumbling block to the success of the programme. Women were still present in midwife obstetric unit (MOU) facilities with no history of EMTCT attendance. Therefore, there is a need for EMTCT service awareness within the community and strengthening collaboration between PHC facilities and community-based teams for bidirectional referral pathways. The use of technology and social media awareness for the provision of EMTCT might increase people’s awareness of these services.

The family-centered approach and male partner involvement have shown an increase in the uptake and retention of EMTCT services among women. However, the male partner, as is taken as the provider for the family, might opt to make an income rather than accompany their partner for their EMTCT services [35,37]. Moreover, the limited space in the facility does not promote male partner involvement. Furthermore, in the African context, male partners who accompany their female partners for EMTCT follow-up are considered weak and jealous. Family involvement in follow-up services encourages women to make informed decisions regarding feeding options and promotes ART adherence [37]. In African families, feeding practices are by an in-law or older family member, as they perceive to know more about infant feeding. Even though guidelines promote exclusive breastfeeding for the first six months, women end up mixing feeding the infants due to fear of HIV disclosure to the family [35,36]. The family-centered approach promotes social and emotional support and empowers women to make informed decisions and adhere to their treatment, EMTCT follow-up, and retention of care.

Community campaigns and awareness enable the uptake of EMTCT services within the community [33]. The literature has proven that the involvement of different stakeholders in the provision of EMTCT services improves their uptake [31,32]. Community stigma and discrimination among people living with HIV can be mitigated through community engagement. Cultural beliefs have a significant impact on the utilization of EMTCT services.

In Africa, women are not allowed to book ANC visits before their pregnancy starts [17]. This has a detrimental impact on the outcome of the program, because women tend to test for HIV late in pregnancy, minimizing the chance of eliminating HIV transmission. Community healthcare workers are required to offer community testing, referral of pregnant women for ANC, provision of family planning, and counselling on ART adherence.

The lack of knowledge of EMTCT services among healthcare workers and patients hinders the success of the programme [29]. Patients rely on healthcare workers for information on EMTCT services. Therefore, it is crucial to provide continuous training on the updated EMTC guidelines. The daily provision of health promotion on EMTCT services promotes its uptake and retention. Even though there has been a significant increase in the number of women who are receiving HIV testing and counselling during pregnancy, barriers such as a lack of information on EMTCT services are still observed. This can be achieved by providing them with accurate information regarding the benefits and risks of these services.

### 4.1. Implication for Intervention

The ramifications of efforts to increase the adoption of EMTCT services in Africa are far reaching. We can save lives and reduce vertical transmission of HIV by increasing the availability and accessibility of EMTCT services. This will have a tremendous effect on the health and well-being of families and communities in Africa. In addition to the obvious health advantages, initiatives to enhance EMTCT service utilization can have a variety of other favorable consequences. For example, they can:

- Reduce stigma and discrimination against people living with HIV

People are less likely to stigmatize and discriminate against those living with HIV when they are better aware of EMTCT services and understand how they work. This can result in greater mental health and well-being for patients with HIV, as well as increased social support.

- Empower women and girls

In Africa, HIV infection affects women and girls disproportionately. Interventions to increase EMTCT service utilization can assist in empowering women and girls by providing them with more control over their health and reproductive decisions. This can result in better health outcomes for young women and girls, as well as increased educational and employment prospects.

- Strengthen health systems

Interventions to promote EMTCT service usage can strengthen health systems by expanding access to care and training healthcare staff. This can lead to better health outcomes for people living with HIV as well as in the general community.

- Promote social cohesion

People are more likely to support HIV prevention and treatment policies and programmes when they are aware of EMTCT services and understand how they work. This can result in a more united and supportive society for persons living with HIV. Therefore, it is critical to involve all relevant stakeholders, including women, adolescents, and family members of women living with HIV, including male partners, nurses, and community leaders.

We can make a significant difference in the lives of individuals living with HIV in Africa by investing in programs to promote the utilization of EMTCT services. We can also create an affluent and healthy future for the continent. We can save lives, decrease stigma and discrimination, empower women and girls, strengthen health systems, and promote social cohesion by making EMTCT services more accessible.

### 4.2. Recommendations

- First, community mobilization and engagement should be promoted through the inclusion of important stakeholders such as HIV-positive women, traditional leaders, and religious leaders. Community mobilization and enlightenment efforts should be made to raise awareness among women about the necessity of access to these services. This can be accomplished through community meetings, radio shows, and outreach. Second, health facilities must have appropriate staffing levels and qualified workers to provide high-quality EMTCT services. This will help to reduce stigma and discrimination against persons living with HIV while also increasing demand for EMTCT services.
- Second, strengthening the health system is required to ensure that EMTCT services are available, accessible, and affordable. This can be accomplished by providing appropriate resources in health institutions, such as employees, equipment, and medication. Furthermore, training health workers on EMTCT guidelines would improve service delivery.
- Third, incorporating EMTCT into MCH would improve access for pregnant women seeking antenatal care. Innovative measures such as task shifting should be developed to improve access to EMTCT services in rural areas. This entails training non-specialist health personnel, such as nurses or midwives, to deliver PMTC services. Finally, improving the utilization of EMTCT services requires strengthening health systems through investment in infrastructure and equipment.
- Finally, to increase resource allocation and coordination efforts, partnerships between governments, non-governmental organizations (NGOs), and international organizations should be expanded. The collaboration will allow for improved strategy formulation and implementation across African areas to boost utilization rates.

### 4.3. Limitations

The review’s limitations focused mostly on measures for improving the utilization of EMTCT services in Africa. This systematic study only examined African articles written in English. Research published on other continents and in other languages was excluded; this research may have influenced the review.

## 5. Conclusions

The effectiveness of EMTCT services can significantly improve MCH outcomes. Increasing the accessibility and affordability of EMTCT treatments is critical for more women to benefit. Healthcare personnel require ongoing training to stay current on protocols and best practices. A holistic strategy that integrates EMTCT with other MCH services offers moms and children comprehensive care. Community health workers and awareness campaigns can help minimize stigma and promote treatment adherence. Family participation and counseling can improve support for HIV-positive mothers. Using mHealth technologies and telemedicine can help enhance service delivery, follow-up care, and treatment adherence, particularly in rural locations. Implementing these strategies can assist in eradicating MTCT and promote healthier futures for moms and children.

## Figures and Tables

**Table 1:** PRISMA-ScR Checklist.

Section	Item	Prisma-SCR Checklist Item	Reported on Page #
Title			
Title	1	Identify the report as a scoping review	1
Abstract			
Structured summary	2	Provide a structured summary that includes (as applicable): the background, objectives, eligibility criteria, sources of evidence, charting methods, results, and conclusions that relate to the review questions and objectives.	1
Introduction			
Rationale	3	Describe the rationale for the review in the context of what is already known. Explain why the review questions/objectives lend themselves to a scoping review approach.	4
Objectives	4	Provide an explicit statement of the questions and objectives being addressed with reference to their key elements (e.g., population or participants, concepts, and context) or other relevant key elements used to conceptualize the review questions and/or objectives.	4
Methods			
Protocol and registration	5	Indicate whether a review protocol exists; state if and where it can be accessed (e.g., a Web address); and if available, provide registration information, including the registration number.	N/A
Eligibility criteria	6	Specify characteristics of the sources of evidence used as eligibility criteria (e.g., years considered, language, and publication status), and provide a rationale.	5
Information sources	7	Describe all information sources in the search (e.g., databases with dates of coverage and contact with authors to identify additional sources), as well as the date the most recent search was executed.	5
Search	8	Present the full electronic search strategy for at least 1 database, including any limits used, such that it could be repeated.	5
Selection of sources of evidence	9	State the process for selecting sources of evidence (i.e., screening and eligibility) included in the scoping review.	5
Data charting process	10	Describe the methods of charting data from the included sources of evidence (e.g., calibrated forms or forms that have been tested by the team before their use, and whether data charting was performed independently or in duplicate) and any processes for obtaining and confirming data from investigators.	6
Data items	11	List and define all variables for which data were sought and any assumptions and simplifications made.	5–6
Critical appraisal of individual sources of evidence	12	If done, provide a rationale for conducting a critical appraisal of included sources of evidence; describe the methods used and how this information was used in any data synthesis (if appropriate).	5–6
Synthesis of results	13	Describe the methods of handling and summarizing the data that were charted.	7
Results			
Selection of sources of evidence	14	Give number of sources of evidence screened, assessed for eligibility, and included in the review, with reasons for exclusions at each stage, ideally using a flow diagram.	14
Characteristics of sources of evidence	15	For each source of evidence, present characteristics for which data were charted and provide the citations.	16
Critical appraisal within sources of evidence	16	If done, present data on critical appraisal of included sources of evidence (see item 12).	7–13
Results of individual sources of evidence	17	For each included source of evidence, present the relevant data that were charted that relate to the review questions and objectives.	7–13
Synthesis of results	18	Summarize and/or present the charting results as they relate to the review questions and objectives.	7–13
Discussion			
Summary of evidence	19	Summarize the main results (including an overview of concepts, themes, and types of evidence available), link to the review questions and objectives, and consider the relevance to key groups.	7–13
Limitations	20	Discuss the limitations of the scoping review process.	21
Conclusions	21	Provide a general interpretation of the results with respect to the review questions and objectives, as well as potential implications and/or next steps.	22
Funding			
Funding	22	Describe sources of funding for the included sources of evidence, as well as sources of funding for the scoping review. Describe the role of the funders of the scoping review.	24

Preferred Reporting Items for Systematic Reviews and Meta-Analyses extension for Scoping Reviews [23].

**Table 2:** Qualities of included studies.

References	Quality of Sample	Control or Comparison Group	Quality of Exposure/Outcome	Follow-Up	Distorting Influences	Reporting of Data	Summary Quality Rating of Study
Pellowski et al.	Adequate	N/A	Adequate	Unclear	adequate	adequate	High
Mutabazi et al.	Adequate	Adequate	Adequate	N/A	Adequate	Adequate	Moderate
Besada et al.	Adequate	Adequate	Adequate	N/A	Adequate	Adequate	Moderate
DiClemente-Bosco et al.	Adequate	N/A	Adequate	Unclear	Unclear	Adequate	High
Oyebode et al.	Adequate	Adequate	Adequate	N/A	Adequate	Adequate	Moderate
Hamilton et al.	Unclear	Unclear	Adequate	N/A	Adequate	Adequate	Moderate
Haika Osaki et al.	Adequate	Adequate	Adequate	N/A	Adequate	Adequate	High
Okal et al.	Adequate	N/A	Adequate	Adequate	Adequate	Adequate	High
Elias et al.	Adequate	Unclear	Unclear	Unclear	Adequate	Adequate	Low
Sam-Agudu et al.	Adequate	Unclear	Adequate	N/A	Adequate	Adequate	Moderate
Helova et al.	Adequate	Adequate	Adequate	Adequate	Adequate	Adequate	Moderate
Malindi F.C	Adequate	Adequate	Adequate	Unclear	Adequate	Adequate	Moderate
Ngoma-Hazemba, A., and Ncama, B. P.	Adequate	Adequate	Adequate	N/A	N/A	Adequate	Moderate
Shroufi et al.	Adequate	Unclear	Adequate	adequate	Adequate	Adequate	High

**Table 3:** Characteristics of included articles.

Authors, Country, and Design	Year	Objectives of the Study	Participants Sample Size	Studies Conclusion
1. Pellowski, J., Wedderburn, C., Stadler, J.A., Barnett, W., Stein, D., Myer, L., and Zar, H.J. Cohort study. Paarl, South Africa	2019	Implementing mother-to-child transmission (EMTCT) prevention in South Africa: outcomes from a population-based birth cohort study in Paarl, Western Cape.	Pregnant women (*n* = 1225)	Although South Africa does not currently meet the criteria for the elimination of MTCT, the study demonstrates that attaining extremely high levels of EMTCT coverage to further reduce transmission rates in high-prevalence regions may be within reach. This may be achieved through retesting, breastfeeding, and ART adherence support, reinforced by improved data surveillance systems.
2. Mutabazi, J.C., Gray, C., Muhwava, L., Trottier, H., Ware, L.J., Norris, S., Murphy, K., Levitt, N., and Zarowsky, C. Qualitative study. South Africa	2020	Integrating the prevention of mother-to-child transmission of HIV into primary healthcare services after AIDS denialism in South Africa: Perspectives of experts and health care workers qualitative study.	Expert = (*n* = 10), frontline health workers (*n* = 10)	While the integration of EMTCT into PHC has been hailed as a success, this research identified ongoing challenges in the integration process for HIV/EMTCT from both the perspectives of experts and FHCWs. Existing issues in bureaucracy and accountability presented barriers to full integration of EMTCT. For FHCWs, concerns of heavy workload and infrastructure constraints, ongoing issues with training, and high staff turnover created challenges in the care of both mother and child.
3. Besada, D., Goga, A., Daviaud, E., Rohde, S., Chinkonde, J. R., Villeneuve, S., … and Doherty, T. Malawi, Uganda	2017	To explore the roles of community cadres in improving access to and retention in care for EMTCT (prevent mother-to-child transmission of HIV) services in the context of EMTCT Option B+ treatment scale-up in high-burden low-income and lower-middle-income countries.	*n*-210 mixed gender	Community cadres provide an integral link between communities and health facilities, supporting overstretched health workers in HIV client support and follow-up.
4. DiClemente-Bosco, K., Weber, A.Z., Harrison, A., Tsawe, N., Rini, Z., Brittain, K., Colvin, C.J., Myer, L., and Pellowski, J.A., Cape Town, South Africa	2022	Empowerment in pregnancy: ART adherence among women living with HIV in Cape Town, South Africa.	Pregnant women (*N* = 30)	These findings suggest that a promising and novel approach to improving ART adherence both during pregnancy and postpartum may focus on taking note of resources as enabling environments, building on existing feelings of agency and self-efficacy, and highlighting both the proximal and distal lifelong achievements associated with adherence that are already deeply entrenched in women’s life goals.
5. Oyebode, T.A., Hassan, Z., Afolaranmi, T., Auwal, M., Shehu, M., Kelechi, N., Oche, A., Sagay, S., Gwamna, J., Okonkwo, P., and Kanki, P. Jos, Nigeria	2021	Improving EMTCT Coverage and Access in Communities with Unmet Needs in Jos, Nigeria by Adopting Task Shifting and Task Sharing Strategies. *European Journal of Preventive Medicine*, 9(3), pp. 83–93.	1200 (pregnant women), 30 (health workers), and 12 (community members)	Addressing HIV/EMTCT gaps will require detailed diagnostics that utilize all appropriate and relevant lenses to analyze the barriers in coverage, access, and uptake of EMTCT services.
6. Hamilton, A. R. L., le Roux, K. W. D. P., Young, C. W., and Södergård, B., Qualitative study. Eastern Cape, South Africa	2020	Exploring the role of a peer mentorship programme in rural EMTCT care in Zithulele, Eastern Cape, South Africa.		Peer mentoring programmes can play an important role in reducing vertical HIV transmission in resource-limited, rural settings by providing participants with education, psychosocial support, and a continuum of care.
7. Haika Osaki1, Saumya S. Sao2, Godfrey A. Kisigo1,2, Jessica N. Coleman2,3, Rimel N. Mwamba2, Jenny Renju4,5, Blandina T. Mmbaga1,4, and Melissa H. Watt2. Tanzania. Qualitative study	2021	To explore how male partner engagement in ANC impacts women’s decision-making to present to ANC and their subsequent experience in ANC in urban health facilities in Northern Tanzania.	13 women and 6 male partners)	Male engagement in ANC can benefit maternal and child health and promote early presentation to EMTCT services.
8. Okal, J. O., Sarna, A., Lango, D., Matheka, J., Owuor, D., Kinywa, E. A., and Kalibala, S. Kenya. Qualitative study	2022	To explore the perspectives of HIV-positive pregnant women attending maternal and neonatal clinic services in Kisumu, Kenya.	27 pregnant women	The fundamental role mobile-phone counselling played in supporting HIV-positive mothers enrolled in ANC by empowering them to address underlying individual, social, and structural factors associated with uptake of services.
9. Elias, M., Mmbaga, E. J., Mohamed, A. A., and Kishimba, R. S. Mwanza region, Tanzania. Cross-sectional study	2017	To examine the predictors of male involvement in EMTCT services in Mwanza Region, Tanzania from the perspective of the mother.	300 women	Male partner involvement is likely to reduce events of gender-based violence.
10. Sam-Agudu NA, Ramadhani HO, Isah C, Anaba U, Erekaha S, Fan-Osuala C, Galadanci H, Charurat M. A Prospective Paired Cohort Study. Rural Nigeria	2017	The MoMent study evaluated the impact of structured vs unstructured PS on postpartum retention and viral load suppression among rural Nigerian women.	497 HIV-positive pregnant women	Structured PS significantly improved postpartum EMTCT retention and viral suppression rates among women in rural Nigeria.
11. Helova A, Onono M, Abuogi LL, Hampanda K, Owuor K, Odwar T, Krishna S, Odhiambo G, Odeny T, Turan JM	2021	To evaluate the acceptability of using cMMs as home-based support for EMTCT services.	*n* = 40 and postpartum women and their partners. *n* = 70 Healthcare worker	Peer support from cMMs during pregnancy through 6 weeks postpartum was associated with improved uptake of critical EMTCT services and health behaviors and was perceived as beneficial for cMMs themselves.
12. Malindi F.C. exploratory sequential mixed method. Limpopo Province, South Africa	2018	To develop a strategy to enhance family-centered interventions for EMTCT sustainability in the selected districts of Limpopo Province,	*N* = mothers of babies between 6 weeks and 18 months *n* = 27 male partners, grandmothers *n* = 15 health care professionals *n* = 27	Involvement of grandmothers and male partners during EMTCT services promotes family involvement.
13. Ngoma-Hazemba, A., and Ncama, B. P. Exploratory descriptive qualitative study. Zambia	2018	To explore the role of community-based volunteers (CBVs) and their perspectives on human immunodeficiency virus (HIV) and infant feeding to gain insights into the implementation of prevention of mother-to-child transmission (EMTCT) interventions at community level	n = 20 HIV-positive mothers, 10 CBVs participated	The role of CBVs in the implementation of EMTCT interventions at community level can be strengthened by improving the training and development of appropriate educational materials that are sensitive to cultural norms and practices in this setting.
14. Shroufi, A., Mafara, E., Saint-Sauveur, J. F., Taziwa, F., and Viñoles, M. C. Bulawayo, Zimbabwe.	2013	To explore, using qualitative methods, the perceptions of relevant stakeholders of the M2M programme.	*N* = 79	M2M programmes offer great potential to empower communities affected by HIV to catalyze positive behavior change. M2M involvement may increase retention in EMTCT programmes.

## Data Availability

Not applicable.
